# Preparedness of Health Care Workers and Medical Students in University Hospital in Krakow for COVID-19 Pandemic within the CRACoV Project

**DOI:** 10.3390/jcm10163487

**Published:** 2021-08-07

**Authors:** Barbara Żółtowska, Ilona Barańska, Katarzyna Szczerbińska, Anna Różańska, Krzysztof Mydel, Wojciech Sydor, Piotr B. Heczko, Estera Jachowicz, Jadwiga Wójkowska-Mach

**Affiliations:** 1 Center for Innovative Therapy, Clinical Research Coordination Center, University Hospital in Krakow, Poland 2-st, 30-688 Krakow, Poland; wojciech.sydor@uj.edu.pl; 2 Laboratory for Research on Aging Society, Department of Sociology of Medicine, Chair of Epidemiology and Preventive Medicine, Medical Faculty, Jagiellonian University Medical College, 31-034 Krakow, Poland; ilona.baranska@uj.edu.pl (I.B.); katarzyna.szczerbinska@uj.edu.pl (K.S.); 3 Chair of Microbiology, Medical Faculty, Jagiellonian University Medical College, 31-034 Kraków, Poland; a.rozanska@uj.edu.pl (A.R.); mbheczko@cyf-kr.edu.pl (P.B.H.); estera.jachowicz@gmail.com (E.J.); jadwiga.wojkowska-mach@uj.edu.pl (J.W.-M.); 4 Deputy Director for Coordination and Development, University Hospital in Krakow, 30-688 Krakow Poland; kmydel@su.krakow.pl; 5 Department of Rheumatology and Immunology, Jagiellonian University Medical College, 30-688 Krakow, Poland

**Keywords:** preparedness, health care workers, medical students, COVID-19, infection prevention and control practices, training

## Abstract

Backgrounds Health care workers’ (HCWs) knowledge of and compliance with personal protective procedures is a key for patients’ and personnel safety. The aim of this study was to assess which factors are associated with higher self-evaluations of training on infection prevention and control (IPC) and higher self-assessment of IPC practices used by HCWs regarding COVID-19 in University Hospital in Krakow, Poland, in January 2021. Material and methods This was an online survey on the preparedness for COVID-19 epidemic of medical/non-medical staff and medical students. Questions included in the survey concerned participants’ socio-demographic characteristics, hospital staff involvement in the training, knowledge about the hand hygiene, and adherence to IPC measures. Knowledge and Performance Index (K&PI) based on selected questions was constructed for to reflect both subjective (self-evaluation) of preparedness and objective IPC knowledge and skills of HCWs participated in the IPC training. Results A total of 1412 health care workers, including 129 medical students, participated in the study. The largest group, 53.6%, was made up of nurses and paramedics. Age of respondents significantly correlated with knowledge of IPC and with K&PI. The mean age of workers with high K&PI was 42.39 ± 12.53, and among those with low, 39.71 ± 13.10, *p* < 0.001. 51% UHK workers participated in IPC training, but 11.3% of physicians, 28.8% of other HCWs, and 55.8% of students did not know the IPC standard precaution. Most participants, 72.3%, felt that they had received sufficient training; however, 45.8% of students declined this. There was no correlation between self-reported preparedness and the K&PI, indicating that self-reported preparedness was inadequate for knowledge and skills. Nurses and paramedics assessed their knowledge most accurately. Participants with low K&PI and high subjective evaluation constituted a substantial group in all categories. Students least often overestimated (23.8%) and most often (9.6%) underestimated their knowledge and skills. **Conclusions** Our study revealed inadequate IPC practice, especially as it refers to the training programme. We confirmed the urgent need of including theory and practice of IPC in curricula of health professions’ training in order to provide students with knowledge and skills necessary not only for future pandemic situations but also for everyday work.

## 1. Introduction

The ability for hospitals to safely provide care to COVID-19 patients requiring hospitalization while maintaining other essential medical services during and after a pandemic, defined as preparedness, is critical for the health of both patients and hospital staff. This definition recognizes that what constitutes “appropriate care”, and the criteria for hospital admission may well change during a pandemic. Hospitals should focus their initial preparedness efforts on limiting the nosocomial spread of the virus to protect the health care workers (HCW) and, thus, maintain a hospital workforce [1]. This is because medical personnel are the most important element in the fight against any epidemic, and unfortunately, they are also the ones most likely to get infected. This is confirmed by data from recent pandemics, e.g., in 2009 (influenza A pandemic, H1N1), the prevalence rate for health care personnel, alone, was more than two times higher compared to controls/comparisons [2]. During the 2014–2016 Ebola outbreak in West Africa, health care workers were 20 to 30 times more likely to be infected than the general public [3]. The previous epidemics of the coronavirus diseases, SARS and MERS, also severely afflicted HCWs. In China, HCWs accounted for 19.60% of total infection cases of SARS, and in Vietnam, most of the probable SARS cases (57.14%) were identified in HCWs [4].

As long as the current pandemic is ongoing, we cannot clearly identify its epidemiological characteristics, but it is likely that the endangering of HCWs will be very similar. Data from March and April 2020 (from UK and USA) indicates that, compared with the general community, front-line health care workers were at increased risk for reporting a positive COVID-19 test; the incidence in in-patient settings was 9.2% vs. 0.3% in general community [5]. Slightly later data, April through August 2020 (only US), however, presents a somewhat different picture: the seropositivity in HCWs was 4.4% (Atlanta, Georgia; Baltimore, Maryland; Chicago, Illinois) and 31.5% in general community (Chelsea, Massachusetts) [6,7]. Furthermore, according to Baker et al., community risk factors are more strongly associated with SARS-CoV-2 seropositivity among health care workers than exposure in the workplace [8]. Perhaps this data illustrates how important continuous education is for infection prevention and how knowledge and skills in infection prevention and control were lacking among health care professionals at the beginning of the epidemic.

According to the World Health Organization (WHO) and European Centre for Disease Prevention and Control (ECDC), the key element of infection prevention and control (IPC) and preparedness for COVID-19 in health care settings is appropriate training on IPC for health care workers and other staff but also for health care workers recruited for surge capacity, including student doctors, student nurses, or retired health professionals [9].

Protecting health care workers is the most important element of infection control in health care settings. Safe staff is essential for patient safety. This study aimed at assessing the knowledge and preparedness of health professionals regarding COVID-19 in University Hospital in Krakow in the beginning of 2021, after the big wave of COVID-19 in Poland in November 2020, when the biggest 14-day rate of reported COVID-19 cases per 100,000 population was 877 (week 46 of 2020). In Poland, from 4 March, when the first case of COVID-19 was detected, until 31 December 2020, a total of 1,322,947 cases were reported, and cumulative incidence per 100,000 was 3483.9 [10]. Included in this number were 87,471 infections of health care workers, 6.6% of all infections [11].

## 2. Methods and Materials

### 2.1. Study Design

This was a non-interventional, uncontrolled, open, single-centre, cross-sectional online survey on the preparedness for COVID-19 epidemic of medical and non-medical staff of University Hospital in Krakow (UHK) and medical students who undergo clinical rotations at UHK.

### 2.2. Setting

The study was carried out in University Hospital in Krakow (UHK), the biggest teaching hospital in Southern Poland, with 39 clinical departments (1310 beds in total), 2 intensive care departments (40 beds), 7 institutes, and 68 out-patient clinics. About 70% of hospital beds are located in the new hospital headquarters, started in October 2019. From March through September 2020, UHK was devoted to COVID-19 patients, only, and from March 2020, it had a universal masking policy and systems for screening, testing (based on the PCR method), and reporting COVID-19 symptoms among staff and patients. Six outbreaks were identified and described: four in October (internal unit, operating theatre, ICU, haematology unit), one in November (surgery unit), and one in December (haematology unit). In total, the outbreaks involved 19 patients and 38 HCWs and cleaning staff.

In 2020, there were 55,140 hospitalized patients (excluding the Hospital Emergency Department). A total of 4998 people worked at the hospital in 2020 (as of 31 December 2020), including 1102 physicians, 1789 nurses, 1668 other health care workers (non-front-line health care workers), and 439 administrative employees, but there was an insufficient daily number of medical and non-medical personnel in COVID departments. From March through December 2020, the medical and non-medical staff and medical students before the start of the clinical rotations and internships were trained irregularly, without any plan, based on demand, ad hoc. According to the documentation of trainings, which were implemented by the infection control team (ICT), training for health care professionals in UHK in the COVID-19 era were carried out according to the same plan, in the same time range; the implementation of the training did not distinguish individual professions, neither in terms of access to training nor topics. Administration employees were trained by the ICT only in the scope of basic IPC, while training on the specificity of care of patients with COVID-19 for this group of employees was available in the remote form, only. The results of audits regarding compliance with hand hygiene and donning and doffing personal protecting equipment (PPE), both in pandemic era and before, were not known.

### 2.3. Study Participants

Participants were investigated between January 4 and 19, 2021. Participation in the study was voluntary. Participants were eligible for enrolment if they met the following inclusion criteria: (1) hospital employee, (2) fifth-year medical student, and (3) voluntary consent to participate in the study. The sole key exclusion criterion was refusal to participate in the study.

Participants were divided into five professional categories based on their level of training in infection prevention and control and according to standards of pre- and post-graduate education:

- medical doctors
- fifth-year medical students
- nurses/midwives and paramedics
- other HCWs (health care assistant, radiology technician, physiotherapist/rehabilitation specialist, dietician, psychologist, social worker, volunteer, cleaning person, diagnostic laboratory technician, registration/patient information desk worker)
- administrative staff

### 2.4. Methods

The study used the modified WHO “Assessment of risk factors for coronavirus disease 2019 (COVID-19) in health workers: protocol for a case-control study” [12]. The questionnaire was modified and translated into Polish and underwent cross-cultural adaptation according to standard procedure. It was piloted in the group of representatives of HCWs in 15–16 December 2020. It contained questions about

(1) socio-demographic characteristics (age, gender, education, profession, and work experience)
(2) hospital staff involvement in the training (participation in general training on the prevention and infections control (IPC); participation in the specific training in the care COVID-19 patients; duration of training on the standard precaution; the form of training in the use of PPE; self-evaluation of the respondent’s preparedness to protect themselves and other staff or patients against infection)
(3) knowledge about the recommended guidance for hand hygiene in healthcare [13]
(4) adherence to IPC measures (whether the respondent is able to properly apply hand hygiene, standard precaution, properly uses PPE)

Information about the study and an invitation to participate was communicated to all University Hospital employees through the hospital’s internal mailing network (January 4 and 11) and the hospital’s PA system (Public Address system). Fifth-year medical students were also invited via email. Only medical faculty and final year students were selected because of their participation in clinical rotations at UHK during the pandemic and completion of the entire pre-graduate medical education. In addition, final year students were invited to volunteer at UHK and other hospitals.

### 2.5. Knowledge and Performance Index

To fully analyse both the subjective, self-evaluation of preparedness and actual, objective IPC knowledge and skills of individuals who participated in the IPC training (all infections, not just COVID-19) conducted at UHK, a composite Knowledge and Performance Index (K&PI) endpoint was introduced, including responses to three questions:

- “Do you know the recommended guidance for hand hygiene in health care?” [13]
- “Do you follow IPC standard precautions when in contact with any patient?”
- “Do you wear PPE when indicated (i.e., surgical masks, goggles/face shields, aprons, coveralls, caps)?”

Each question had only one response that indicated a high level of knowledge and skill, and these were: “five moments of hand hygiene”, “I always follow standard precaution”, and “I always use personal protective equipment according to the exposure assessment”. A respondent was classified as having a high Knowledge and Performance Index if they provided the above responses to the questions.

### 2.6. Statistical Analysis

#### Sample Size

The following assumptions were used to calculate the optimal sample size: general sample size (total number of employees): 5000; expected prevalence: 0.5; margin of error: 3%; confidence level: 95%. Based on this, the optimal sample size was calculated to be 880 individuals. Knowing the employment structure of the hospital (22% of employees are doctors, 36% nurses, 33% other HCWs, and 9% administrative staff), 193 doctors, 317 nurses, 290 other HCWs, and 80 administrative staff should be included in the study. There were 1412 participants in the study: 1283 hospital employees (25.8% of all) and 129 fifth-year students majoring in medicine (55.8% of all). Among front-line participants there were: 124 physicians (11% of all doctors in the hospital); 757 nurses and paramedics (42% of all); 233 others HCWs (20% of all), and 169 of administrative staff (18% of all).

**Figure 1:**
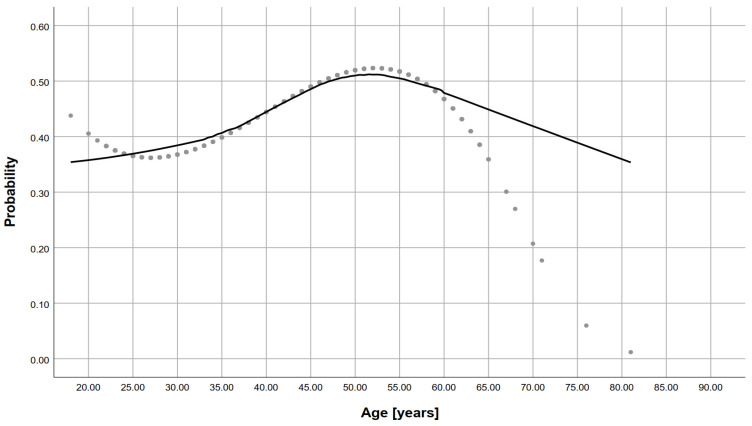
Impact of age on the Knowledge and Performance Index: self-reported compliance in several IPC behaviours. Figure presents scatter plot with LOWESS function fitting to show the relationship between age and knowledge index (K&PI). Since K&PI is a binary variable, the plot shows the probability of high knowledge calculated from the logistic regression model (dependent variable: knowledge (low/high), independent variables: age + age^2 + age^3.

The characteristics of the sample by occupation are presented in Table 1. Differences in response distributions between groups were assessed using the chi-square test. Next, univariable and multi-variable analyses were conducted to determine what factors are associated with self-evaluation of IPC training (S&E training; Table 2 and Table 3) and adherence to IPC measures (Table 2 and Table 3) among hospital staff. For this purpose, respondents’ answers to these two questions (i.e., self-evaluation of IPC training and correct application of IPC procedures) were recorded by combining the categories “definitely yes” and “rather yes” as yes and “definitely no”, “rather no”, and “I don’t know” as no. Univariable analyses used chi-square test and multi-variable analyses used logistic regression. Results in Table 3 are presented as odds ratio (OR) and 95% confidence interval (95%CI). For the chi-square tests, a multiple comparison with Bonferroni correction was performed for tables larger than 2 × 2. Responses from those who did not participate in any IPC training (*n* = 233) were excluded from the analyses on self-evaluation of IPC training. Additionally, those individuals who responded, “I do not know the IPC standard precautions”, to the question regarding the form of training and duration of training were excluded from the analyses regarding self-assessment of training and proper use of infection control procedures (*n* = 211 and *n* = 286, respectively). This reduced the number of administrative staff; hence, this occupational group was excluded from univariable and multi-variable analyses. Due to the presence of collinearity between age and work experience, only the variable age was entered into the multi-variable models. Figure 1 is a scatter plot with LOWESS function fitting to show the relationship between age and Knowledge and Performance Index (K&PI). Since the variable K&PI is a binary variable, the plot shows the probability of high knowledge calculated from the logistic regression model (dependent variable: knowledge [low/high]; independent variables: age + age^2 + age^3). An alpha level of <0.05 was adopted for the analyses to ascertain the statistical significance of the effect. Analyses were performed using the IBM SPSS Statistics 26 for Windows package (IBM SPSS Statistics, IBM Corporation, Chicago, IL, USA).

### 2.7. Ethics Approval and Consent to Participate

The study was conducted according to the guidelines of the Declaration of Helsinki and approved by the Bioethics Committee of the Jagiellonian University (protocol code 1072.6120.353.2020, date of approval 16 December 2020). All data entered into the electronic database and analysed in this study has been anonymized.

## 3. Results

### 3.1. Descriptive Characteristics of Study Population

A total of 1412 people participated in the study, including 129 fifth-year medical students. Nurses, midwives, and paramedics constituted the largest group (*n* = 757, 53.6%). Age of respondents significantly correlated with knowledge of IPC. The mean age of those with high K&PI was 42.39 ± 12.53, and among those with low K&PI, 39.71 ± 13.10 (t = −3.586, *p* < 0.001). The probability of high K&PI increases with age and peaks between 50 and 55 years of age and decreases in older employees. A total of 722 (51%) UHK workers who completed the questionnaire participated in IPC training, which was significantly more common among the front-line HCWs (91.9% physicians, 96.3% nurses and paramedics), slightly less common among others (76.4%), and least common among students and administration staff (57.4% and 49.7%, respectively) (Table 1), but 11.3% of physicians, 28.8% of other HCWs, and 55.8% of students did not know the IPC standard precaution. In contrast, only 3.4% of nurses and paramedics and 49.7% of administrative staff admitted to not knowing the standard precaution. Considerably fewer participants of the study declared participation in specific training in the care COVID-19 patients. According to the respondents, the most common form of training was theoretical (*n* = 558, 39.5%) or theoretical with some elements of practice (*n* = 592 41.9%) (Table 1).

A significant proportion of the participants (72.3%) assessed that they had received sufficient training, but students were significantly more likely (45.8%) to say that their training was inadequate, and they were significantly more likely (22.5%) to say that they were unable to assess whether they were sufficiently trained and prepared to work in a pandemic situation. This was confirmed by the responses to the next question, where a significant number of students did not know how to properly use PPE (19.4%) or could not assess their skills in this area (26.4%) (Table 1).

Medical students assessed their training as sufficient less often (41.9%) compared to other occupations. This is also confirmed by age analysis, where people aged 37 and more were significantly more likely to indicate adequate training. A similar trend can be seen in relation to years of service, where only those working more than 5 years were significantly more likely to respond affirmatively (82.5%). Participants of COVID-19 training felt significantly more confident (88.5%), as did those whose training lasted 2 h or more (90.5%) (Table 2). Those who demonstrated a high level of knowledge and skills in IPC were significantly more likely to confirm that they had received adequate training (84.9%). Multi-variate analysis of self-evaluation of preparedness for care of patients with COVID-19 (front-line participants and other HCWs, only) indicated that it was significantly higher among those aged 37 or older (compared with those aged 18–36) and non-front-line participants (compared with physicians). Participation in specific training in the care COVID-19 patients and IPC training in a remote format mode with practice components impacted preparedness scores among staff (OR, 2.8; 95% CI, 2.03–4.35; and OR, 2.8; 95% CI, 1.87–4.10, respectively), but there was no correlation between self-evaluation of preparedness and the K&PI (OR, 1.4; 95% CI, 0.98–2.09; *p* = 0.066), indicating that self-evaluation of preparedness was inadequate for knowledge and skills (Table 3).

In terms of applied IPC practices, medical students (45.7%), those aged up to 36 (17.1%), and men (18.9%) were significantly more likely to indicate lack of skills. Participation in the specific training in the care COVID-19 patients (98.2%) practical and theoretical training (97.2%) were associated with higher assessment of one’s own skills in terms of IPC practices. Those with high self-assessment of IPC skills scored significantly higher on the K&PI (97.4) (Table 2). In multi-variable analysis, occupation was found to be significantly associated with higher self-assessment of IPC skills, but nurses/midwives and paramedics and other HCWs rated their skills significantly higher compared to physicians (OR, 4.2; 95% CI, 1.60–11.15; and OR, 4.9; 95% CI, 1.00–24.17, respectively). Participants of COVID-19 training were more likely to believe that they could correctly use IPC procedures compared to those who did not receive such training. However, using the K&PI showed a lack of relationship between actual and declared skills (OR, 2.1; 95% CI, 0.95–4.85; *p* = 0.066) (Table 3).

### 3.2. Knowledge and Performance Index

**Figure 2:**
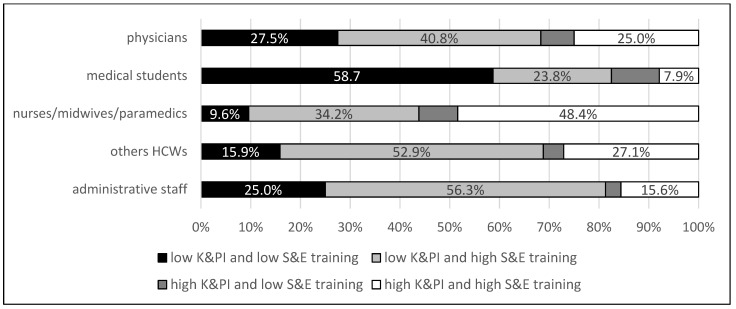
Consistency analysis of subjective (self-evaluation of IPC training) and objective responses based on Knowledge and Performance Index. The graph shows an analysis of the compliance of the survey participants’ responses regarding their self-evaluation of IPC (S&E) and objective, actual knowledge and skills in the field of infection prevention and control (K&PI, Knowledge and Performance Index). High S&E training means that the respondent strongly agrees or agrees that he or she has been sufficiently trained in infection prevention and control, and low indicates strong disagreement, disagreement, or inability to judge it. A respondent was qualified as having a high K&PI index if he or she correctly indicated that there are five moments of hand hygiene and always follows the IPC standard precaution and always uses personal protective equipment when indicated. The analysis was performed by occupational groups.

Analysing the convergence between the subjective self-evaluation of IPC training and the objective index (K&PI), nurses, midwives, and paramedics assessed their knowledge most accurately (48.4%), and only one-fourth of physicians assessed their knowledge correctly (Figure 2). Participants with low K&PI and high self-evaluation of preparedness constituted a substantial group in all categories (34.2% of nurses, midwives, and paramedics; 40.8% of physicians; and more than half of other HCWs and administration staff) (Figure 2). Students, consistent with previously presented data, rarely overestimated their knowledge and skills (23.8%), but this group also had the highest number of individuals who underestimated their knowledge and skills (9.6%). The Knowledge and Performance Index taking three variables into account was checked and supplemented by adding a question where the correct answer was that the best choice for hand hygiene is alcohol-based hand rub. Then, only 11.2% of the respondents, i.e., 84 people out of 747 who were involved in direct patient care, scored high on the supplemented Knowledge and Performance Index.

## 4. Discussion

Multi-variable analyses did not confirm a statistically significant association between self-evaluation of preparedness for COVID-19 patient care and actual knowledge and skills in basic elements of infection prevention and control (hand hygiene, ICP standard precautions, or use of personal protective equipment).

In our study, representatives of various health care professions rated their preparedness to work with COVID-19 patients and to protect themselves and others from infection to varying degrees. They also rated participation in training to varying degrees. Overall, medics were more likely to participate in training and more likely to confirm good preparedness, but among them, physicians were the group that participated in training less frequently than others, and the training was more likely to be short (<2 h) and remote. This was closely related to the K&PI scores achieved by the different professions, where only one-third of physicians scored high, while the score for nurses/midwives and medics was almost two times higher. The better knowledge of infection control and prevention rules among nurses than physicians was also confirmed by other authors. Al Qadire M et al. confirmed this phenomenon with regard to procedures related to the prevention of catheter-related bloodstream infections [14], and Chang et al. confirmed it in regard to hand hygiene compliance at critical points of care [15]. An unsatisfactory level of physicians’ knowledge of hand hygiene was reported also by Wałaszek et al. in a questionnaire study at another teaching hospital in the south of Poland [16].

The results for different professional groups were different, despite the fact that the studied hospital infection control team trained every medic to the same extent, regardless of their profession; it is therefore probable that non-physicians used the training to a much greater extent, while physicians who opted for short and/or remote training gained much less knowledge and skills. Fortunately, one-third of the surveyed physicians report not feeling properly trained, meaning they are open to education. On the other hand, a significant part—over four-fifths—of the remaining HCWs considering themselves well-trained and able to apply IPC practices obtained a poor result measured by K&PI, because less than two-thirds of nurses/midwives and paramedics and one-third of other HCWs had a high result. Therefore, an unsatisfactory level of knowledge in the field of IPC was associated with a sense of good preparation and good training. There is probably no room for these groups to acquire new knowledge or implement new procedures in the field of IPC, which may be worrying, because the discrepancies between the knowledge and practice of selected procedures, including hand hygiene, among doctors and nurses without significant differences, were also shown in the study Wałaszek et al. [17]. Unluckily, unsatisfactory knowledge, practice, and perception of hand hygiene and PPE procedures in Poland are probably related not only to sub-optimal forms and organization of continuous training but are strongly conditioned by the specific organizational culture of Polish hospitals [17].

More than four-fifths of administrative staff lacked knowledge of IPC, e.g., they did not know what the standard IPC precautions and “the 5 moments for hand hygiene” are, although they declared a high level of correctly applied IPC practices. Similar results were obtained by Zafar et al., where physicians and nurses scored higher on COVID-19 knowledge compared to non-clinical hospital staff [18]. Significant improvement in knowledge was observed after training. The study concluded that non-clinical staff, an essential part of healthcare, must be educated, and access to information must be a cornerstone of clinical practice—which also seems necessary in the studied hospital, especially since it is a teaching hospital.

It was found that almost half of the medical students who do their internships and have clinical rotations at the hospital did not attend or did not remember that they attended either the training on IPC or the specific training in the care COVID-19 patients. Unfortunately, this was confirmed by the fact that they rated their preparedness to work under pandemic conditions, and thus biological threat, very poorly. More than half of the students did not know the standard precaution; hence, more than 80% of the students achieved a low rating of knowledge and skills. In the case of training prior to participation in clinical classes, the results of the present study are more disappointing than the results of a study on the knowledge of medical students at the same university seven years ago, in which less than one-fifth of the students (22.9%) declared lack of training in hospital hygiene, and even more, almost one-third (28%), declared lack of training in hand hygiene [19]. In the cited study, almost half of respondents declared that the training preceding professional practice did not cover the topic of occupational exposure to biological agents [19]. The fact that students are poorly prepared to practice medicine in biohazardous conditions is very alarming. This is crucial, because the teaching standards of medical, nursing, and midwifery students currently lack the information about infection prevention and control, which results in such sub-optimal levels of self-reported PPE and IPC training, both in Poland and in other countries [20]. Unfortunately, in the course of teaching, the attention of both teachers and students is focused on infectious diseases, which, unfortunately, is not synonymous with IPC. There are positive examples of a modern approach to implementation of infection surveillance including elements of protection of HCWs from biological threats, such as the rules concerning physicians working in the State of New York, where all licensed healthcare professionals are required to receive training on infection control and barrier precautions every four years [21]. In Poland, post-graduate training also lacks content on infection prevention and control with the exception of rarely chosen specialties such as medical microbiology and infectious diseases, while every physician, regardless of future specialization, will have to deal with patients with infectious diseases or colonized with highly transmissible or epidemiologically important pathogens, not only SARS-CoV-2.

Fortunately, students were most often among those surveyed aware of their deficiencies in knowledge and skills—almost 60% showed both low self-evaluation and objective confirmation of this in their answers, so they are the group that is probably most open to education. Hence, it is necessary to make changes to the curricula in order to utilize this potential for knowledge acquisition and to prepare students for possible future participation in anti-epidemic activities so that they are able to provide medical care under conditions that protect patients and staff from transmission of infections. The goal of these changes is to better prepare the physicians for necessary public health activities and to acquire the knowledge and skills to safely treat and isolate infected patients. Such changes are necessary, since studies in the USA have indicated, for example, that physicians were not prepared for their work at the beginning of the current pandemic and found it very difficult to adapt to necessary changes in their work performance under emergency conditions, as confirmed by the higher incidence of COVID-19 among physicians than among other professions [22]. Moreover, in China, physicians acquired the necessary skills only during the pandemic [23]. According to Margaret Fu-chun Chan Fung, medical schools are the most important partners of all public health organizations working to reduce global epidemic events [24]. There have been several recent publications in the medical literature about the need for changes in medical student education. One survey of students found that their overall level of knowledge regarding hand hygiene, safe disposal of sharps, and use of personal protective equipment was low, inadequate, and left them vulnerable to infections while attending health care facilities [25,26]. According to Ashcroft et al., a specific training program is needed for medical students recruited to assist. Medical students receiving appropriate training may have an important role in pandemic control, but the core curriculum in current education does not provide this type of preparation [27]. Reports on the sources of medical students’ information regarding COVID-19 are disturbing. In Khasawneh’s study, students cited social media (83.4%) and internet search engines (84.8%) as the most common source of information, with less reliance on medical search engines (64.1%) [28]. The results of various studies, mainly pre-pandemic, confirm that training with elements of practice is much more beneficial, but training during a pandemic is very difficult, as it is organized under pressure of time, while the need for distancing is incompatible with the fact that the best solution for clinical teaching is face-to-face teaching [29]. Additionally, Byrnes et al. showed that about one-fifth of medical students surveyed now believe that the COVID-19 pandemic will affect their choice of specialty. The main reasons are insufficient opportunities to explore specialist knowledge or a lack of such opportunities [30]. On the other hand, COVID-19 highlighted the positive impact of online education. According to O’Doherty et al., it is teachers who are largely responsible for effective online learning and should understand that their own approach to teaching will, in fact, need to change to adjust the online environment [31].

Unfortunately, it is possible that a relatively frequent declaration of lack of knowledge of infection protection principles and lack of skills to apply them in practice by medical students results not only from insufficient knowledge acquired during studies but also from confusing confrontation with HCWs’ practice and unsatisfactory compliance with recommendations. In the study carried out by Różańska et al., greater clinical experience of students (measured both by length of service and duration of practice) correlated with more frequently declared observation that the personnel did not follow recommended hand hygiene practices [19]. The observation of overestimated self-assessment of individuals aged 55 and older is consistent with the study by Rosiński et al., who found a significant decrease in the standard precaution adherence index among health care workers with 35 or more years of work experience [32], which is characteristic of this age group. It is difficult to assess whether it is the effect of the knowledge from many years ago, when hand hygiene and precautions were slightly different than now, or the effect of burnout and occupational fatigue, but in the light of shaping the attitudes of students and young HCWs [33,34] the educational effort should also be dedicated for this group. A nationwide study conducted by Abulebd et al. indicated that clinical simulations are the preferred form of training [35]; unfortunately, in the studied hospital, the dominant form of training was remote learning and simulations, and practical teaching constituted a negligible part of the training—which also resulted from the situation of working under the pressure of increasing epidemiological threat.

Unfortunately, a confirmation of the problems associated with adequately preparing staff for pandemic conditions and inadequate staff health surveillance is the fact that the only six outbreak investigations described in UHK involved staff (medical and non-medical) with two times more cases than patients: 19 vs. 38 individuals. In addition, the lack of outbreak investigations from March through September indicates a possible inadequate investigation or surveillance of HCWs infections system, since, given the highly contagious nature of SARS-CoV-2 [36], it is reasonable to assume that outbreaks of infection also occurred earlier (before October 2020) but were not described and analysed, so it is not known how many staff became ill and under what circumstances they were exposed to the virus.

### Strengths and Limitations

The results presented here are not only the first study of preparedness outside the English-speaking countries conducted on a large sample of respondents, in the largest university hospital in Poland, where clinical training is regularly conducted, but, due to the nature of the survey, it was not conducted on a random sample. An assessment of the representativeness of the data for the staff of the entire hospital indicates an overrepresentation of nurses. Due to the intensity of staff responsibilities in relation to the care of COVID-19 patients, no objective survey (test) of knowledge was conducted but only a survey of subjective assessment of the training of the respondents.

## 5. Conclusions

Infection control and prevention in modern medicine is not only prevention of infectious diseases but is dominated by highly transmissible or epidemiologically important pathogens, such as SARS-CoV-2, or multi-drug-resistant microorganisms. The problem must be recognized by medical professionals, health care administrators, and politicians, but most importantly by the academic community. It is difficult to ensure the safety of medical staff and trainee students in a hospital in a pandemic scenario. In the hospital studied, infection prevention and control activities were inadequate, and the training system was especially lacking. None of the professional groups received satisfactory training, but gaps in knowledge among medics regarding IPC were especially true for physicians and students participating in clinical classes. Thus, it is mandatory to include theory and practice of infection control in curricula of the final years of medical studies and various health professions’ studies in order to provide HCWs with knowledge and skills necessary not only for future pandemic situations but also for contact with pathogens in everyday work.

## Figures and Tables

**Table 1:** Characteristics of the study group.

Characteristics of the Study Group/Profession	Total N = 1412	Physicians N = 124	Medical Students N = 129	Nurses/Midwives and Paramedics N = 757	Other Health Care Workers N = 233	Administrative Staff N = 169	*p* Value *
Sex, *n* (%)							
Female	1241 (87.9)	87 (70.2)	82 (63.6)	734 (97.0)	208 (89.3)	130 (76.9)	<0.0001
Age, *n* (%)							
18–36	524 (38.4)	73 (58.9)	129 (100.0)	223 (29.5)	72 (30.9)	45 (26.6)	<0.0001
37–55	655 (46.4)	40 (32.3)	0 (0.0)	402 (53.1)	113 (48.5)	100 (59.2)	
56 or older	215 (15.2)	11 (8.9)	0 (0.0)	132 (17.4)	48 (20.6)	24 (14.2)	
Work experience, *n* (%)							
Less than 12 months	126 (8.9)	5 (4.0)	41 (31.8)	46 (6.1)	21 (9.0)	13 (7.7)	<0.0001
1–5 Years	334 (23.7)	37 (29.8)	86 (66.7)	107 (14.1)	54 (23.2)	50 (29.6)	
6 Years or longer	952 (67.4)	82 (66.1)	2 (1.6)	604 (79.8)	158 (67.8)	106 (62.7)	
Have you attended specific training in the care COVID-19 patients? *n* (%)							
yes	722 (57.5)	70 (58.3)	17 (15.3)	520 (69.7)	99 (51.0)	16 (18.8)	<0.0001
no	534 (42.5)	50 (41.7)	94 (84.7)	226 (30.3)	95 (49.1)	69 (81.2)	
Have you attended infection prevention and control training (all infections, not only COVID-19)? *n* (%)							
yes	1179 (83.5)	114 (91.9)	74 (57.4)	729 (96.3)	178 (76.4)	84 (49.7)	<0.0001
no	223 (16.5)	10 (8.1)	55 (42.6)	28 (3.7)	55 (23.6)	85 (50.3)	
How much cumulative IPC training to standard and additional transmission-based precautions have you received at your health care facility? *n* (%)							
<2 h	908 (64.3)	88 (71.0)	53 (41.1)	580 (76.6)	136 (58.4)	51 (30.2)	<0.0001
2 h or more	218 (15.4)	22 (17.7)	4 (3.1)	151 (19.9)	30 (12.9)	11 (6.5)	
I don’t know what IPC standard precautions are	286 (20.3)	14 (11.3)	72 (55.8)	26 (3.4)	67 (28.8)	107 (63.3)	
Was the IPC training on standard and additional transmission-based carried out remotely or practical? *n* (%)							
Only remotely	558 (39.5)	58 (46.8)	47 (36.4)	298 (39.4)	91 (39.1)	64 (37.9)	<0.0001
Only practical	51 (3.6)	4 (3.2)	6 (4.7)	28 (3.7)	7 (3.0)	6 (3.6)	
Both, practical and remotely	592 (41.9)	53 (42.7)	9 (7.0)	420 (55.5)	88 (37.8)	22 (13.0)	
I don’t know what IPC standard precautions are	211 (14.9)	9 (7.3)	67 (51.9)	11 (1.5)	47 (20.2)	77 (45.6)	
Do you think you have received enough training to protect yourself and others from infection? *n* (%)							
yes	1021 (72.3)	81 (65.3)	41 (31.8)	623 (82.3)	185 (79.4)	91 (53.8)	<0.0001
no	236 (16.7)	39 (31.5)	59 (45.8)	97 (12.8)	22 (9.5)	19 (11.3)	
I don’t know	155 (11.0)	4 (3.2)	29 (22.5)	37 (4.9)	26 (11.2)	59 (34.9)	
Can you correctly apply IPC practices? N (%)							
yes	1252 (88.6)	109 (87.9)	70 (54.3)	742 (98.0)	219 (94.0)	112 (66.3)	<0.0001
no	55 (3.9)	6 (4.8)	25 (19.4)	4 (0.5)	6 (2.6)	14 (8.3)	
I don’t know	105 (7.4)	9 (7.3)	34 (26.4)	11 (1.5)	8 (3.4)	43 (25.4)	
Are you familiar with the “hand hygiene moments” recommended by WHO for daily practice in health care? *n* (%)							
I don’t know them	112 (7.9)	9 (7.3)	19 (14.7)	9 (1.2)	16 (6.9)	59 (34.9)	<0.0001
Yes, all 3	96 (6.8)	8 (6.5)	2 (1.6)	20 (2.6)	32 (13.7)	34 (20.1)	
Yes, all 4	33 (2.3)	4 (3.2)	3 (2.3)	6 (0.8)	9 (3.9)	11 (6.5)	
Yes, all 5	943 (66.8)	87 (70.2)	90 (69.8)	621 (82.0)	104 (44.6)	41 (24.3)	
Yes, all 6	228 (16.1)	16 (12.9)	15 (11.6)	101 (13.3)	72 (30.9)	24 (14.2)	
Knowledge and Performance Index *n* (%)							
High *n* = 537 (45.1)	537 (45.1)	38 (31.7)	22 (17.5)	418 (56.3)	53 (31.2)	6 (18.8)	<0.0001
Low *n* = 654 (54.9)	654 (54.9)	82 (68.3)	104 (82.5)	325 (43.7)	117 (68.8)	26 (81.3)	

Abbreviations: IPC, infection prevention and control; PPE, personal protective equipment; WHO, World Health Organization. * Differences in response distributions between groups of occupations were assessed using the chi-square test.

**Table 2:** Factors associated with self-evaluation of IPC training and self-assessment of proper applied IPC practices—only front-line participants and other HCWs, without administrative staff.

Characteristics of the Study Group	Do You Think You Have Received Enough Training to Protect Yourself and Others from Infection?		*p* Value *	Can You Correctly Apply IPC Practices?		*p* Value *
	No, *n* = 226 (20.6%)	Yes, *n* = 869 (79.4%)		No, *n* = 103 (8.3%)	Yes, *n* = 1140 (91.7%)	
Occupation, *n* (%)						
Physicians	37 (32.5)	77 (67.5)	<0.0001	15 (12.1)	109 (87.9)	<0.0001
Medical students	43 (58.1)	31 (41.9)		59 (45.7)	70 (54.3)	
Nurses/midwifes and paramedics	123 (16.9)	606 (83.1)		15 (2.0)	742 (98.0)	
Other HCWs	23 (12.9)	155 (87.1)		14 (6.0)	219 (94.0)	
Age [year], *n* (%)						
18–36	136 (34.5)	256 (65.5)	<0.0001	85 (17.1)	412 (82.9)	<0.0001
37–55	73 (13.9)	453 (86.1)		14 (2.5)	541 (97.5)	
56 or older	17 (9.7)	158 (90.3)		4 (2.1)	187 (97.9)	
Sex, *n* (%)						
Female	189 (19.0)	804 (81.0)	<0.0001	78 (7.0)	1033 (93.0)	<0.0001
Male	37 (36.3)	67 (63.7)		25 (18.9)	107 (81.1)	
Participation in specific training in the care COVID-19 patients, *n* (%)						
Yes	80 (11.5)	615 (88.5)	<0.0001	13 (1.8)	693 (98.2)	<0.0001
No	136 (38.5)	217 (61.5)		76 (16.3)	389 (83.7)	
Form of general training, *n* (%)						
Only remote	130 (28.6)	324 (71.4)	<0.0001	29 (5.9)	465 (94.1)	<0.0001
Practical and remote or practical	60 (10.3)	525 (89.7)		17 (2.8)	598 (97.2)	
Duration of IPC training, *n* (%)						
<2 h	162 (20.0)	648 (80.0)	<0.001	36 (4.2)	6 (2.9)	<0.0001
2 h or more	19 (9.5)	180 (90.5)		821 (95.8)	201 (97.1)	
Knowledge and Performance Index, *n* (%)						
high	77 (15.1)	434 (84.9)	<0.001	14 (2.6)	517 (97.4)	<0.0001
low	136 (25.9)	389 (74.1)		76 (12.1)	552 (87.9)	

Abbreviations: IPC, infection prevention and control. * *p*-value for the chi-square test.

**Table 3:** Multi-variable analysis, self-assessment of preparedness for patient care, and use of infection protection procedures—only front-line participants and other HCWs, without administrative staff.

Study Group/Profession	Self-Assessment of Preparedness for Patient Care			Use of IPC Practices		
	OR	95% CI	*p*	OR	95% CI	*p*
Sex, female vs. male	1.77	0.95–3.23	0.071	0.88	0.34–2.26	0.794
Age						
18–36	Ref			Ref		
37–55	2.47	1.62–3.77	<0.0001	1.81	0.68–4.79	0.233
56 or older	3.19	1.69–6.01	<0.0001	2.07	0.44–9.72	0.355
Occupation						
Physicians	Ref			Ref		
Medical students	1.35	0.60–3.04	0.473	0.44	0.16–1.19	0.106
Nurses/midwifes and paramedics	1.11	0.63–1.94	0.720	4.22	1.60–11.15	0.004
Others HCWs	3.34	1.4–7.98	0.007	4.92	1.00–24.17	0.050
Participation in specific training in the care COVID-19 patients, yes vs. no	2.97	2.03–4.35	<0.0001	3.59	1.55–8.33	0.003
Form of general training, remote with practice vs. remote	2.78	1.87–4.10	<0.0001	1.25	0.57–2.75	0.584
Duration of IPC training, 2 h or more vs. <2 h	1.51	0.87–2.63	0.141	0.83	0.30–2.25	0.711
Knowledge and Performance Index, high vs. low	1.42	0.98–2.09	0.066	2.15	0.95–4.85	0.066

Abbreviations: IPC, infection prevention and control.

## Data Availability

The datasets generated or analyzed during this study are available and can be obtained, at request, from Jadwiga Wojkowska-Mach (e-mail: Jadwiga.wojkowska-mach@uj.edu.pl) on reasonable enquiry.
